# Survey of wastes management status of Khayyam industrial estate in Neyshabur City (Northeastern Iran) in 2017

**DOI:** 10.1016/j.mex.2019.02.018

**Published:** 2019-02-26

**Authors:** Hossein Alidadi, Ali Akbar Mohammadi, Alia Asghar Najafpoor, Aliakbar Dehghan, Sima Zamand, Vahid Taghavimanesh

**Affiliations:** a Department of Environmental Health Engineering, School of Public Health, Mashhad University of Medical Sciences, Mashhad, Iran; b Department of Environmental Health Engineering, Neyshabur School of Medical Sciences, Neyshabur, Iran

**Keywords:** Application of survey of wastes management status, Waste, Wastes management, Khayyam industrial estate, Neyshabur, Iran

## Abstract

The extent of environmental pollution caused by wastes in industrial estates has attracted the attention of scientific and executive resources of the world to the correct disposal and recycling of these materials. This study is a cross-sectional descriptive study which was conducted in the Khayyam industrial estate of Neyshabur in 2017. To survey the management of industrial wastes status, based on the fieldwork, different days were selected in each week to complete the questionnaire designed by the Iranian Environmental Protection Agency. The completion of questionnaires was started from a specific path daily. Finally, the collected data were entered into the SPSS 18 software and were analyzed to determine the wastes management status of the industrial estate. Excel software was used to draw the charts. The total industrial wastes produced in Khayyam industrial estate were 3555 tons per year, waste per capita was 2177.9 g per day per worker. Most existing industries in Khayyam industrial estate were food and pharmaceutical, which include 43.94%. Recycling (79.55%) and burial (14.39%) were the first and second levels of final disposal of industrial wastes. The results of this study show that the wastes management of Khayyam industrial estate has a relatively acceptable situation. Unfortunately, the waste dumping in the open space can lead to the generation of moisture and the release of pollutants to the environment and cause pollution of water and soil through leakage as well as damage to workers. Proper control and correct management are essential for maintaining the health and protection of the environment.

## Graphical abstract

**Figure:**
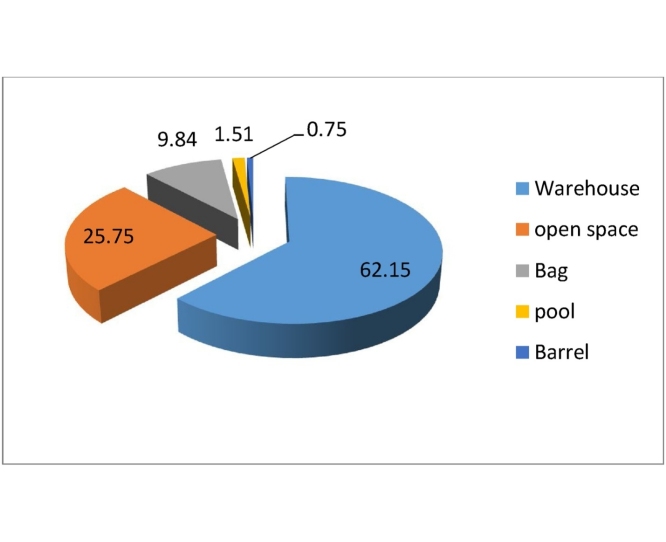

**Table:** 

Subject Area	Environmental Science
More specific subject area:	Wastes Management
Method name:	Application of Survey of Wastes Management Status
Name and reference of original method	Ajero C, Chigbo U. A Study on the Evaluation of Industrial Solid Waste Management Approaches in Some Industries in Aba, South Eastern Nigeria. West African Journal of Industrial and Academic Research. 2012;4(1):1–10
Resource availability	The data are available with this article

## Method details

The establishment and development of the industries pursue the economic and social development and seek the goals such as increasing domestic production, creating the jobs and improving quality of life are among the developmental indicators of each country [1]. In addition, it is categorized as development indicators of each country [2]. A lot of raw materials are used to produce new products that are led to generate a lot of wastes [3]. Nowadays, the main source of hazardous wastes produced in the world is industrial activities [2] and are problematic as a part of byproducts generated in industrial activities [4]. These wastes have severe effects on environmental factors such as water, soil and air and affect the health and safety of workers in the community [5]. The extent of environmental pollution caused by wastes in industrial estates has attracted the attention of scientific and executive resources of the world to the correct disposal and recycling of these materials [6,7]. Industrial estates are places that have been prepared and built for industrial activities [8]. These places play an essential role in the development of a country [9]. If they are correctly utilized, they will facilitate the achievement of urban goals and economic developments [10]. But industrial estates generate large amounts of Non-consumable wastes that are released into the environment [8]. Industrial waste refers to all wastes obtained from the industrial activities [11]. These wastes are considered as hazardous wastes due to their specific characteristics [12]. Hazardous wastes are considered due to the lack of biodegradability, cumulative effects and destructive impacts on human health and living organisms [13]. Nowadays, the amount of hazardous wastes generated by the industries in the world, is estimated to be about 370 million tons per year [14]. Many industries produce new contaminants which some of them are carcinogenic and toxic [15,16]; Thus, industrial wastes have become a serious issue in the world [17]. These material, as byproducts, are very problematic [4]; So that the industrial estates produce 1.1 billion tons of industrial wastes annually [18]. These wastes include 3 types; solid, semi-solid and liquid materials [17] and consist of food, ash, special and hazardous waste, paper, plastics, glass and etc [19]. Therefore, in order to protect the community health and to reduce the destructive effects of industrial wastes, the precise recognition of the industry, quantity, quality and waste management is necessary; this depends on type of industry, equipment, facilities, management, and personnel from viewpoint of quantity and, it is divided into six categories consist of inorganic wastes, oily materials, organic materials, non-decayed materials, low-risk materials and miscellaneous materials from viewpoint of quality [5]. Effective control and correct management of industrial wastes are important for the health, environmental protection and management of natural resources [20]. Today, the efficient management of industrial wastes due to the large amount and variety is a serious challenge for industries [21]. In developed countries such as France, Netherlands, Germany, and Japan, there is a trustworthy database of industrial wastes, and this issue has a great importance in these countries; but waste management has not grown in developing countries and these countries have many problems in this filed [22]. On the other hand, inappropriate management of industrial waste creates many problems for humans and environment; therefore, proper control and management and comprehensive planning and research are essential to protect health and environment [23]. In recent years, the quantitative and qualitative identification and the analysis of industrial waste status have been carried out in some provinces [24]. Therefore, due to the lack of accurate information about the management of industrial wastes in Iran and lack of the studies about the industrial waste in Khayyam Industrial Estate, the present study aims to survey the wastes management produced by industries and to reduce the problems caused by industrial wastes in 2017.

## Materials and methods

**Fig. 1:**
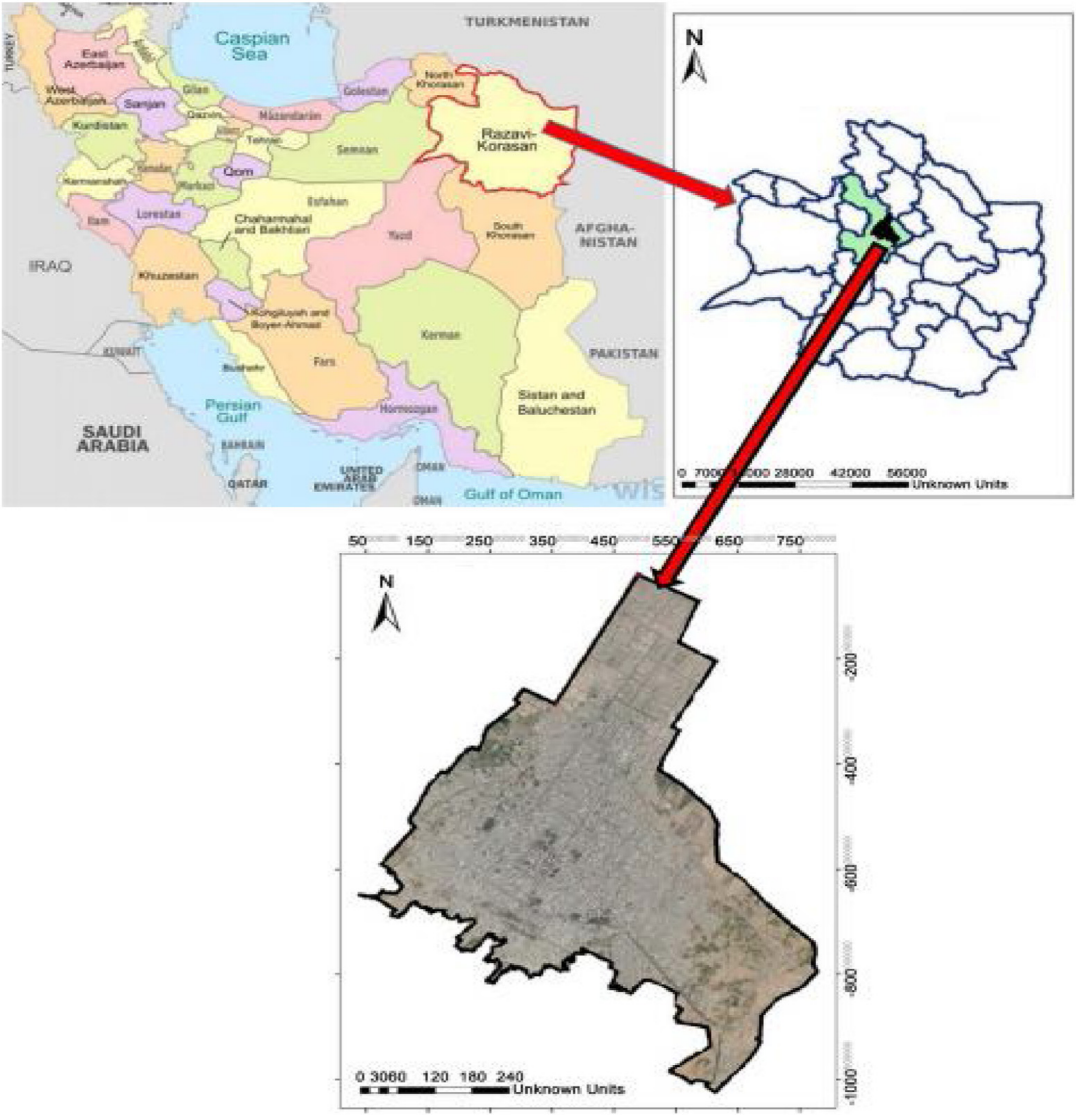
Location of study area in Neyshabur city, Khorasan Razavi province, Iran.

Neyshabur is one of the most important cities of Iran in terms of the population, cultural background, tourism, industrial and historical centers in the northeast of the country (Khorasan Razavi province). Neyshabur is located at 36° 10′ north latitude and 58° 50′ east longitude and the distance between this city with Sabzevar and Mashhad is 100 km and 120 km, respectively. Neyshabur has relatively warm weather in the summer and cold weather in winter. The highest and lowest temperatures of this city are 40.5 °C and −20 °C, respectively. The average annual temperature in this city is reported to be 14.8 °C and the average precipitation is 365.8 mm/year. Neyshabur with 451,780 persons is the second most populous city in Khorasan Razavi province (Fig. 1). This study is a cross-sectional descriptive study which was conducted in Khayyam industrial estate of Neyshabur in the year 2017. This industrial estate has located on the 20th km of Neyshabur-Mashhad road and is one of the largest industrial estates in the province and has good conditions in terms of facilities. In addition, its area is 246 ha and has 144 active industries.

A large number of active units generate a lot of wastes and these wastes should be correctly managed because they can be harmful to the environment and humans. There are three methods to study the production, composition, and quantity and quality of produced wastes, including the empirical method, the use of questionnaires and available control data. Since there is no reliable industrial information and a consistent database of waste management systems in the relevant departments, the standard questionnaire designed by the Iranian Environmental Protection Agency was used for all active industries in this study. The questionnaire had 25 questions and 4 sections including basic information, quality, quantity and wastes management [4,5]. Based on the fieldwork, different days were selected in every week to complete the questionnaire which was started from a specific path daily. For this purpose, a questionnaire was completed for each industry by referring to industrial units. Completion of these questionnaires was accomplished by the persons, who had sufficient knowledge about waste, such as environmental health experts in the industries, or the managers of the industries. According to the classification of the Iranian Environmental Protection Agency, the industries are divided into 10 groups consisting of food and pharmaceutical, textile, wood and cellulose, paper, plastic and chemical, non-metallic, metal, machinery, cosmetics, and electronic industries.

In order to better assess the quantitative part of the questionnaire, 10 samples were selected from the largest industrial units of each group, based on the classification of the Environmental Protection Agency; so that the percentage of the existing components could be matched and compared with the information of the questionnaires. According to this, at the end of the working day, at the first, the wastes were completely mixed with a shovel or other device, and a sample was then taken from the point that the mixing took place well and was lack of the coarse components. It was divided into three equal parts and, finally, one kilogram of waste was taken from one part of the samples [25]. Then, it was weighed with a 0.01 g precision lab scale (Sartorius, GE812). The samples were analyzed physically and the components in each sample, such as paper, plastic, and others were determined. In order to provide more safety, during separation and weighing operations, the personal protective equipment such as glove, and mask were used. In this study, the amount of profitability and economic analysis of the recyclable components of the wastes were calculated and analyzed based on the price of the Iranian Waste Management Organization. In this method, at the end of the work, the weight of each component of the waste was obtained and the percentage of each component and the economic value were calculated according to the total weights of the industrial wastes produced. Finally, the collected data from the questionnaire and the samples were entered into the SPSS 18 software and were analyzed to determine the wastes management status of the industrial estate. Excel software was used to draw the chart.

## Conclusion

The results of this study show that the wastes management of Khayyam industrial estate has a relatively acceptable situation. Nevertheless, the significant results of this study were the application of waste reduction equipment in a small number of waste management processes used in industries, which its importance has recently supported and emphasized in many developed countries. Therefore, in this case, this problem should be resolved through appropriate management solutions. In addition, this can have a significant share in the recycling products, which has left the production cycle due to the lack of appropriate knowledge and technology. Meanwhile, environmental pollution has a lot of significant losses for industrial producers from the viewpoint of economics. Therefore, it is necessary that the industrial wastes pass the codified plans, the process of volume reduction and separation, and then dispose of correctly. Another remarkable result is the mixing of industrial wastes with domestic wastes, which have many inappropriate consequences, especially for recycling issues. According to the results of this study, if all the wastes produced are introduced to the recycling cycle, it will lead to a profit of $ 640,613. Also, recycling can help to protect the environment. Therefore, accurate planning, correct training and implementation of the recycling process can lead to the use of the industrial wastes. According to the obtained results, the percentage of generated wastes was matched to the taken samples. Plastic, paper, ferrous metals and organic materials were the most produced wastes in Khayyam Industrial Estate. Generally, the quantity and quality of waste generated in industries depend on many factors such as generation rate, product type, quality of raw materials and devices, process type, product and raw material value, management, staff skill levels, and the climate of the waste collection environment.

## Discussion

**Fig. 2:**
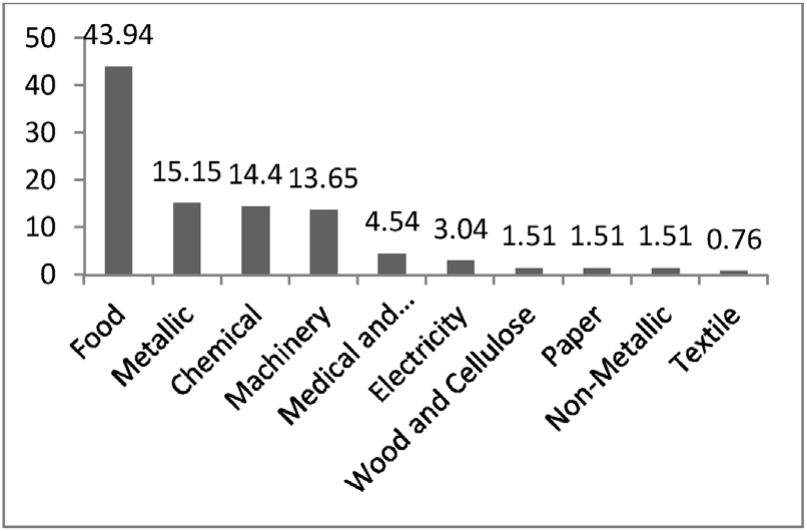
Types of industries in Khayyam industrial estate.

Fig. 2 shows the type of present industries in the Khayyam Industrial Estate., According to the figure, most existing industries were the food and pharmaceutical industries (43.94%). The metal industries (15.15%) and chemical (14.4%) with 14.4% were other largest industries in this estate. In addition, textile industries (0.76%) were identified to be the least industries in Khayyam industrial estate with 0.76%.

**Fig. 3:**
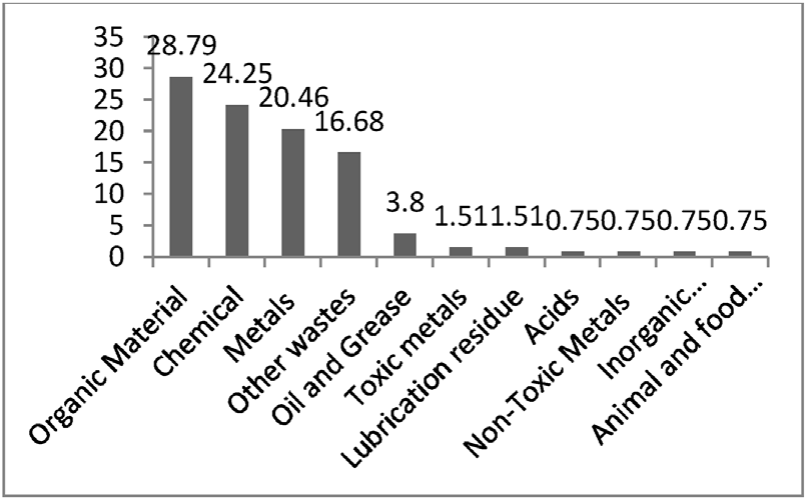
Percentage of industrial wastes qualitively.

The results showed that the total number of personnel in Khayyam Industrial estate was 4472 persons and the waste per capita was 2177.9 g per day per worker. The volume of produced wastes was 376.09 cubic meters. The results also show that 87.2% of the industries separated their wastes in the origin and the separation in the origin is not carried out in12.8% of industries. Fig. 3 shows produced wastes in Khayyam industrial estate, qualitatively. Accordingly, organic matter (28.79%) was the most industrial waste in the industrial estate. Chemical (24.25%) and metals (20.46%) were in the next places, respectively. Oils and greases (3.8%), toxic metals (1.51%), lubricants (1.51%), acids (0.75%), inorganic compounds (0.75%) and animal and food debris (0.75%) were hazardous wastes produced in Khayyam Industrial estate.

**Fig. 4:**
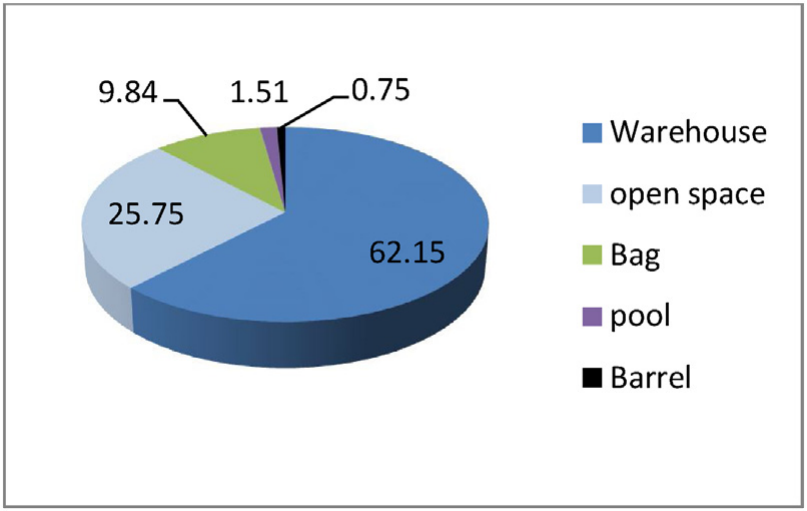
Temporary storage of industrial wastes in Khayyam industrial estate.

Results showed that the commonly used methods for temporary storage of the waste in the industries are the warehouse (62.15%) and the open space (25.75%). The bag (9.84%), pool (1.51%), and barrel (0.75%) were the other used methods for temporary storage of the industrial wastes in Khayyam industrial estate (Fig. 4).

According to the information in this study, 59.11% of industrial wastes were disposed on monthly basis, 39.39% on weekly basis and 0.75% on daily basis and 0.75% on annual basis.

Results showed that 84.09% of the wastes were disposed by the industrial owners, and the private sector was responsible for disposal of 15.91% of industrial wastes.

**Fig. 5:**
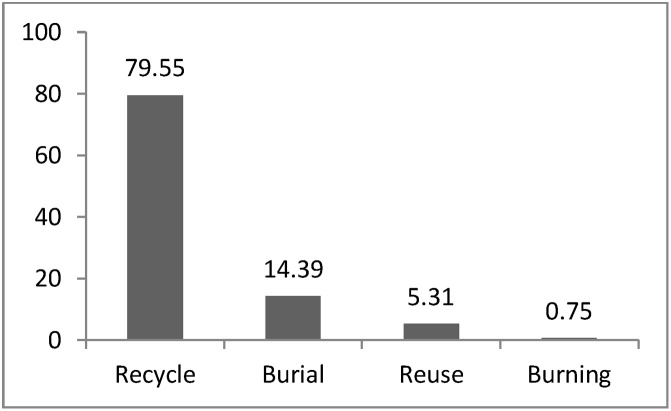
final method for disposal of industrial wastes in Khayyam industrial estate.

**Fig. 6:**
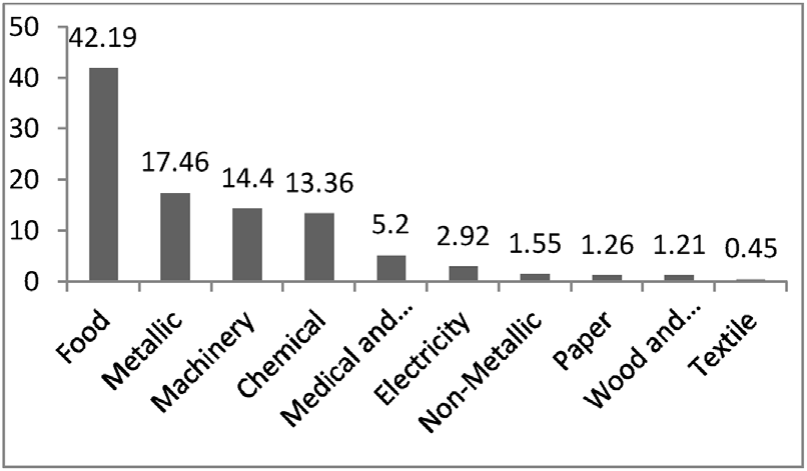
Percentage of generated wastes by industries.

Fig. 5 shows that the methods used for final disposal of industrial wastes in the industrial estate were recycling (79.55%), burial (14.39%) and reuse (5.31%). respectively. In this study, only 0.75% of wastes were burned. Based on Fig. 6, the total generated wastes in Khayyam Industrial estate were 3555 tons per year and the largest waste producer in the industrial estate was identified to be the food industries (42.19%). Metal industries, with 17.46% and machinery, with 14.4%, were in the next rank, respectively. The lowest waste producers were textile industries (0.45%) and wood and cellulose (1.21%).

**Fig. 7:**
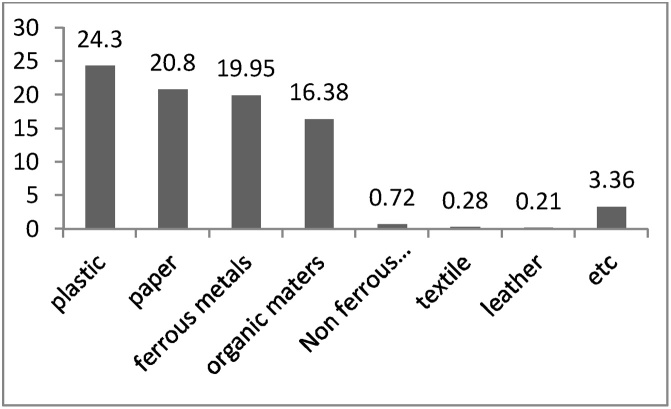
Percentage of wastes components in Khayyam Industrial Estate.

Fig. 7 shows the components of produced wastes in Khayyam industrial estate. Accordingly, plastic (24.3%) was the largest wastes produced by industries. Ferrous metals (20.8%) and paper (19.95%) were in the next ranks, respectively. The percentage of other components including organic matter, non-ferrous metals, textiles, and leather were 16.38%, 0.72%, 0.28%, and 0.21%, respectively.

**Fig. 8:**
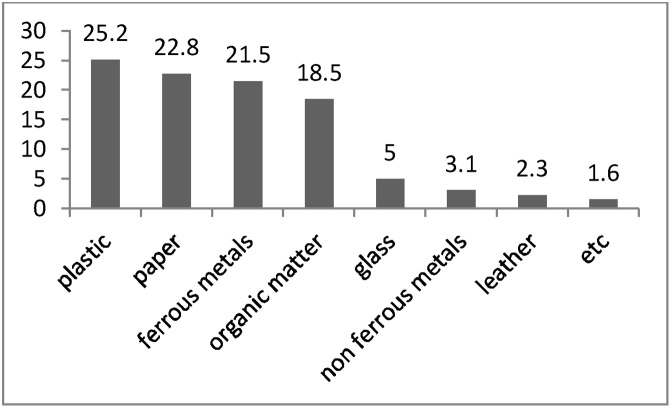
Result of the physical analysis of samples.

Fig. 8 shows the results of 10 samples taken from the largest industries of each group to better examine the quantitative part of the questionnaire. Accordingly, the most produced wastes were detected to be the plastics (25.2%) and, paper (22.8%). Ferrous metals (21.5%) and organic matter (18.5%) were placed in the next ranks, respectively. Glass (5%), non-ferrous metals (3.1%), and leather (2.3%) were also other produced wastes. Other wastes were 1.6% (It contained acids, oils and greases, animal and food waste).

In this study, food industries (43.94%) was the most available industries in Khayyam industrial estate, but, in the study conducted by Charmondusit in Thailand in 2012, it was observed that most deployed industries in the industrial estate were Electronic Industries (36% of all industries) [26]. In another study which was conducted in Kuwait in 2008, the most industries were observed to be the textile-based industries, accounting for 47% of total [27]. In this study, per capita waste generation per worker was 2177.9 g per day and wastes volume were 376.09 cubic meters; our results are not agreed with the study conducted in Yazd industrial estate in 2010 in which the volume of produced wastes were 519.2 cubic meters [28]. Moreover, Shahbazi and Sofyanian reported that per capita waste per worker in the Isfahan Industrial estate was 9650 g per day [24].

In this study, 87.2% of industrial units separate their wastes at the origin of generation; considering the problems caused by lack of origin separation, especially in the field of collection, transfer and recycling, it is a remarkable sign for existence of good education and correct planning by managers and excellent cooperation of the private sector, In the study conducted by Shahbazi and Sofyanian, it was observed that only 10% of industrial units in Isfahan Industrial estate had a separation program in the origin of generation, which was very low compared to Khayyam Industrial Estate [24]. According to the obtained results, only 9.7% of Khayyam industrial estate wastes were considered as hazardous in terms of quantity; these kinds of wastes should be exactly investigated because they are associated with inappropriate impacts on water, soil and air and workers’ health. Karami et al., in their study conducted in the industrial area between Tehran-Karaj in 2015, was determined that the total amount of hazardous wastes was 12% [4]. The results showed that the main approach for temporary storage of industrial wastes was to maintain the wastes in a suitable warehouse, which was estimated 62.15%; this method can facilitate the recycling and transportation of the wastes. Also, it was observed that 25.75% of industries keep their produced wastes in open space. Ajero et al., in their study in Nigeria, observed that 55% of industries keep their wastes in open space; this seems to be inappropriate and can pose various environmental risks [29]. Hasanvand et al. reported that 66% of studied industries in Quchan keep their wastes in open space for temporary storage [30]. The waste dumping in the open space can result in the generation of moisture and the release of pollutants to the environment, which it can cause the pollution of water and soil through leakage as well as damage to workers. According to the results, 59.11% of industrial wastes were disposed on a monthly basis, which indicates the weakness in transportation or management of the industries. The results of the study conducted by Bemani et al. revealed that 57.2% of industrial wastes are disposed on daily basis [28]. Thus, the daily disposal of the wastes generated should be implemented to reduce the major risks and problems caused by inappropriate monthly disposal. According to the results, industrial wastes (84.09%) are mainly disposed of by the industries owner and 15.91% of the waste is disposed by the private sector, which disposes of the wastes, as a contractor, under the supervision of the industries. Abedinzadeh and Monavari introduced the inefficiency of the transportation system and waste disposal as one of the main weakness in the industrial estate of Rasht [12]. The results of the analysis show that wastes recycling (79.55%) and burial (14.39%) are the first and second options for waste disposal. Roudbari et al. revealed that 51.2% of produced wastes in Shahrood industrial estate is recycled, which is similar to the results of this study [31]. In another study, Hasanvand et al. reported that the recycling rate in Quchan city is only 16% and 80% of the wastes are buried; this is indicative of the lack of planning by the industries and related authorities [30]. The wastes burning in the open space is led to the dispersion of polluting gases, such as dioxins and has a lot of environmental hazards. Fortunately, only about 0.75% of the industrial wastes are burned in Khayyam industrial estate, which should be immediately managed as possible as.

The results in Fig. 6 show that the total amounts of produced wastes in the Khayyam Industrial Estate were 3555 tons per year; in other words, 7.9 tons per day of waste are produced. Among the various industries in the studied estate, 42.19% of the total wastes generated, were related to food industries and these industries were considered as the largest waste producer; a large number of these industries is considered as its main reason. Metal (17.46%) and machine industries (14.40%) were in next ranks, respectively. On the other hand, textile industries had the lowest portion (0.45%) in industrial wastes production because of the low number of this type of industry in Khayyam industrial estate. In the study conducted in the Bu-Ali Industrial estate of Hamadan by Binavapour et al., the total amounts of generated waste were 3632 tons per year [5]. Furthermore, Mbuligw reported that the total produced wastes in Tanzania were 39,000 tons per year, which was a significant value [32]. According to the results, plastics were the most part of industrial wastes residuals, which indicates the use of this material in various industries, e.g., packaging. Binavapoor et al. observed that the highest produced wastes in Hamadan were chemicals (34.74%) and organic material (15.44%), respectively [5]. In another study conducted in Pakistan in 2014, plastics were accounted for 25% of the industrial wastes, which is similar to this study [33]. These differences or similarities in types and amounts of produced waste can be attributed to differences in the nature of existing industrial units, the increase of the recycling and reuse, minimization of industrial wastes, training and deployment of environmental ISOs [4].

## Conflict of interest

The authors of this article declare that they have no conflict of interests.
